# To go or not to go? Biological logic gating engineered T cells

**DOI:** 10.1136/jitc-2021-004185

**Published:** 2022-04-04

**Authors:** Rebecca C Abbott, Hannah E Hughes-Parry, Misty R Jenkins

**Affiliations:** 1 Immunology Division, Walter and Eliza Hall Institute of Medical Research, Parkville, Victoria, Australia; 2 Department of Medical Biology, The University of Melbourne, Parkville, Victoria, Australia; 3 Institute for Molecular Science, La Trobe University, Melbourne, Victoria, Australia

**Keywords:** immunotherapy, receptors, chimeric antigen, cytotoxicity, immunologic, cell engineering

## Abstract

Genetically engineered T cells have been successfully used in the treatment of hematological malignancies, greatly increasing both progression-free and overall survival in patients. However, the outcomes of patients treated with Chimeric Antigen Receptor (CAR) T cells targeting solid tumors have been disappointing. There is an unmet clinical need for therapies which are specifically designed to overcome the challenges associated with solid tumors such as tumor heterogeneity and antigen escape. Genetic engineering employing the use of biological logic gating in T cells is an emerging and cutting-edge field that may address these issues. The advantages of logic gating include localized secretion of anti-tumor proteins into the tumor microenvironment, multi antigen targeting of tumors and a potential increase in safety when targeting tumor antigens which may not be exclusively tumor specific. In this review, we introduce the concept of biological logic gating and how this technology addresses some of the challenges of current CAR T treatment. We outline the types of logic gating circuits and finally discuss the application of this new technology to engineered T cells, in the treatment of cancer.

## Introduction

Chimeric antigen receptor (CAR) T cell immunotherapy has initiated a new class of therapies, redirecting a patient’s T cells to recognize and destroy malignant cells. CARs are modular, synthetic receptors consisting of an antigen binding domain (usually a short-chain variable fragment (scFv)) joined via a hinge to a transmembrane domain, costimulatory domain (commonly CD28 or CD137/4-1BB; each with different kinetics^1^) and a signaling domain (CD3ζ). CAR expression on the T cell redirects the effector T cell to a user-defined antigen, releasing the constraints of T cell receptor (TCR)-mediated antigen recognition of peptide in the context of Major Histocompatibility Complex (MHC).

Conventional CAR T cell therapy has led to great improvements in patient survival and reducing disease burden in patients with hematological malignancies, with the pan-B cell marker CD19 most commonly targeted.^2^ However, it is clear from the published results of multiple clinical trials that more research is required to enhance CAR T cell efficacy against solid tumors.^3–6^ There are many challenges in the application of CAR T cells to solid tumors including the immunosuppressive tumor microenvironment, efficient trafficking into the tumor and extensive tumor heterogeneity (as reviewed.^7 8^). Therefore, alternate engineering of CAR T cell therapies for solid tumors will be required to improve efficacy (table 1).^9^.

**Table 1:** Advanced chimeric antigen receptor designs such as SUPRA, UNI, Pro and Split have been applied to enhance control over engineered cells

Structure	Description	Published example
SUPRA	Split, universal and programmable CAR system (SUPRA), the scFv and main CAR receptor are separate and become joined with a leucine zip.	Cho *et al*^76^
UNI	Universal receptor as the common base, which is inactive. The binding domain of the injectable, interchangeable scFv binds to the uniCAR allowing full CAR T function.	Cartellieri *et al*,^77^ Ma *et al*^78^, Tamada *et al*,^79^ Urbanska *et al*^80^
Probody/ masked CAR	Therapeutic receptor in which the active binding domain of the antibody is in an inactive form, which can become activated by tumor-associated proteases.	Han *et al*^81^
Split	Antigen recognition motif is dissociated from the signaling motif of the CAR or signaling and costimulation domains are separated.	Rodgers *et al*,^82^ Kudo *et al*,^83^ Lanitis *et al*,^84^ Kloss *et al*^85^

CAR, chimeric antigen receptor; CRS, Cytokine Release Syndrome; scFv, short-chain variable fragment.

As a living drug, CAR T cells can persist and proliferate, consequently, mechanisms to control their number and/or function are desirable. The earliest attempts to control CAR T cell function focused on either permanent T cell destruction by employing ‘suicide switches’^10 11^ or degradation of the CAR protein itself, by targeting with PROteolysis Targeting Chimeras (PROTACs).^12^ Suicide switches are small molecule mediated circuits which, after the administration of a drug or monoclonal antibody, results in the rapid destruction of infused T cells.^10^ This type of engineering is particularly beneficial when rapid removal of T cells is required, such as when patients are experiencing acute adverse events like excessive release of proinflammatory cytokines, cytokine release syndrome (CRS), on-target off-tumor toxicity or graft vs host disease.^11 13^ PROTACs induce the degradation of the CAR protein itself, instead of the entire cell, a system pharmacologically mediated by promoting ubiquitination of the CAR protein, tagging it for degradation by the proteasome.^12^ PROTACS were proposed to have the potential to enhance the safety of CD19-targeting CAR T cells,^12^ by allowing for the CAR protein to be degraded, leaving the T cell still functional. Clinically, this system could be deployed in patients who present with symptoms of CRS; enabling treatment of the cause, rather than the symptoms of the condition. Considering the significant time and financial cost in generating patient-specific CAR T cell therapies, the complete and irreversible destruction of all circulating CAR T cells by suicide switches or even the CAR protein may not be sustainable technologies in the field.

Neither genetic suicide switches nor PROTACs address the issue of on-target off-tumor toxicity. Additionally, there is an inevitable time delay between a patient displaying symptoms of toxicity, and CAR degradation or CAR T cell apoptosis, therefore a de novo designed, self-regulating system is of high clinical relevance.

## Advantages to employing logic gated systems in biology

**Figure 1:**
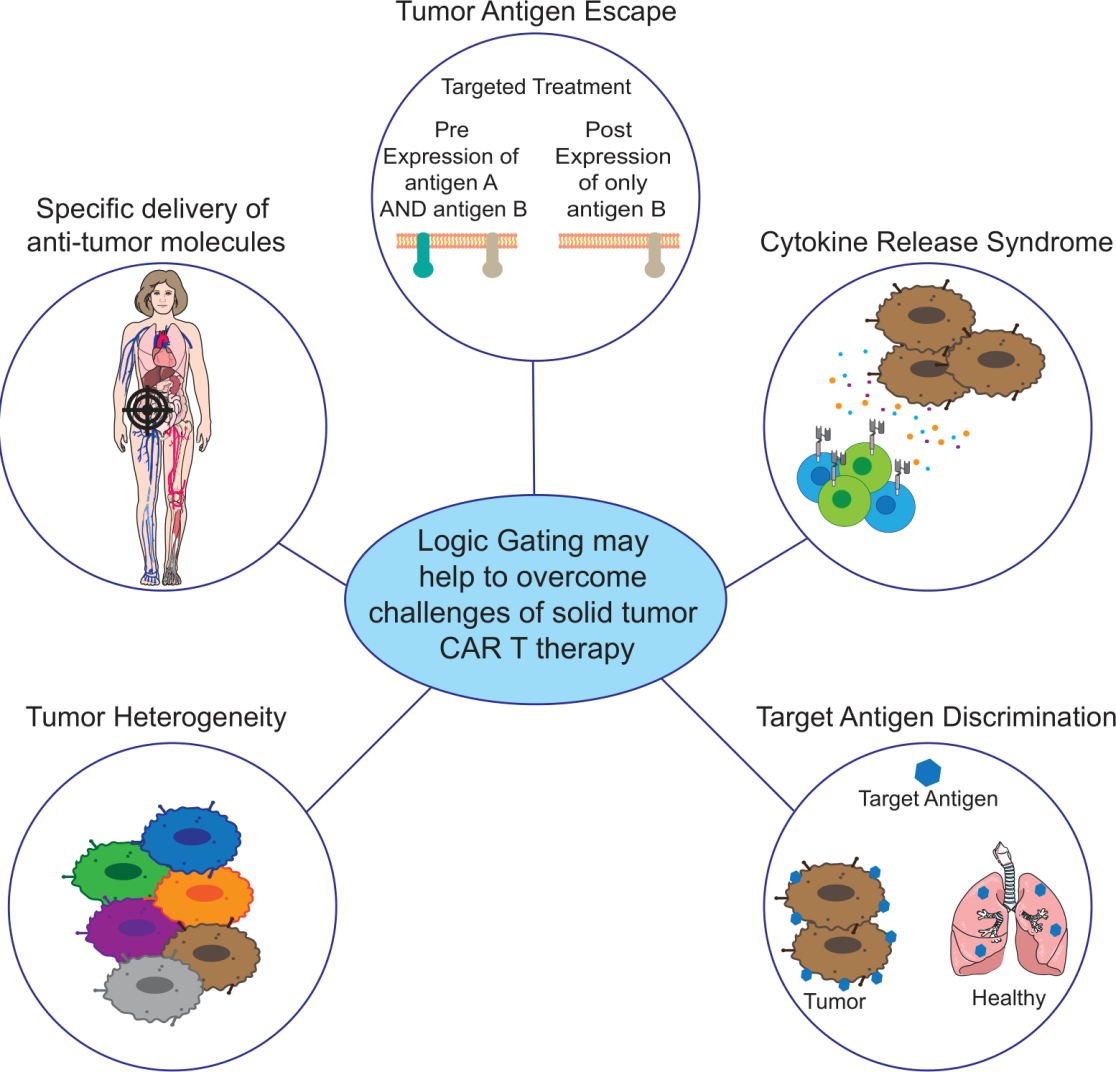
Employment of logic gating strategies may address some of the considerations associated with current CAR T cell therapy for solid tumors. Localized production of antitumor molecules via logic gating systems reduces systemic adverse effects in patients and directly delivers these molecules where they are needed. Multiantigen targeting with logic gating has the potential to address both tumor heterogeneity and tumor antigen escape by targeting more of the tumor cells with a single treatment, with the goal of more complete elimination of tumor cells to protect against relapse. Immune responses such as CRS or off tumor toxicity could potentially be managed with logic gated production of anti-inflammatory or immune modulating molecules within the tumor microenvironment, inhibiting CAR function against healthy tissues. Tumor microenvironment gated expression of functional molecules such as cars is a method to enhance discrimination capabilities between healthy and malignant tissue. CAR, chimeric antigen receptor.

As the field moves to discover more tumor specific antigens (TSA), and we untangle the relationships between avidity and antigen level expression on tumor vs healthy tissue, more sophisticated and specific methods of regulating T cell responses using logic gating are being developed.^14–16^ Biological logic gating has the potential to enhance the efficacy of cell-based therapies by enabling the direct and localized modulation of the tumor microenvironment. There are many forms of logic gating, the most common being AND-, OR- and NOT-. User-defined programs induced by logic gating can include the expression of cytokines, chemokines, biologics, additional CARs or inhibitory signaling. The advantages of logic gating systems can be summarized into three main categories: (1) localized expression of anti-tumor mediators; (2) reducing the likelihood of tumor antigen escape compared with conventional CAR T therapy and combating tumor heterogeneity and; (3) modulating immune responses such as CRS or protection of healthy tissue via enhancing capacity for antigen discrimination (figure 1).

### Localized delivery of anti-tumor molecules using logic-gating

One advantage of applying logic gating to biological therapies is to trigger localized secretion of ant-tumor molecules, within the tumor microenvironment. Logic gated molecule release results in the highest concentration being delivered directly at the tumor site, rather than the molecule being sequestered in other tissues. Second, the specific delivery of molecules may allow for the application of a wider range of antitumor molecules, which may be unsuitable for systemic delivery. Additionally, well-established non-logic gated systems such as nuclear factor of activated T cells (NFAT)-dependent (T cells redirected for antigen-unrestricted cytokine-initiated killing (TRUCKs;fourth generation CAR T cells)^17^ (reviewed^18 19^) and chemically controlled systems^20 21^(reviewed^22^) can also be employed for site specific delivery of modulating factors.

There are several non-specific biological agents which have demonstrated efficacy in promoting anti-tumor responses. Interleukin-12 has potent anti-tumor efficacy and has shown to synergise with CAR T cell therapy in glioblastoma,^23^ yet is systemically toxic.^24^ Similarly, HER-2-specific CAR T cells secreting the dendritic cell growth factor Fms-like tyrosine kinase 3 ligand (Flt3L) induced epitope spreading in host T cells against non-HER-2 tumors in a transgenic OT-I murine system, particularly with the addition of immune adjuvants.^25^ CD19-CAR T cells engineered to express CD40L also facilitated the recruitment of endogenous immune cell populations in a syngeneic immunocompetent mouse model.^26^ These agents may be promising candidates for gated expression in the tumor microenvironment to enhance immune responses.

However, there must also be stringent pre-clinical evaluations of such therapies. The final doses delivered at the tumor site may remain unknown or be under limited control and therefore the half-life of these molecules must be determined to ascertain potential toxicity risks. Despite these considerations, there are numerous possibilities in the types of treatments which could be delivered via logic gating.

### Limiting antigen escape and combating tumor heterogeneity

Antigen escape is the process of specific downregulation of a tumor expressed protein resulting from selective pressure exerted by targeted therapy or the outgrowth of pre-existing antigen negative clones. While antigen escape has been observed in hematological malignancies,^2 27^ ^28^ this remains a greater challenge for solid tumors, due to the increased tumor antigen heterogeneity. Tumor heterogeneity has been observed both between patients (interpatient heterogeneity) and within patient tumors (intratumoral heterogeneity).^29^ Patient tumor sampling and biomarker testing, particularly before enrolment in relevant clinical trials, have demonstrated activating mutations present in proteins such as KRAS,^30^ BRAF^31^ and EGFR^32^ within tumors and between patients. Tumor heterogeneity is a contributing factor to resistance against mono-targeted therapies,^33^ and with advancing technology this type of tumor analysis has been further enhanced by single-cell technologies.^34^ Such technology enables monitoring of a patient’s tumor over the course of targeted therapy to allow earlier identification of target antigen downregulation and antigen escape and potential re-evaluation of the treatment plan. The phase I EGFRvIII CAR T cell trial (*NCT02209376*) allowed patients to participate in the trial with EGFRvIII positivity ranging from 6%–96% (calculated by RNA sequencing of the number of EGFRvIII reads/ total EGFR and EGFRvIII reads).^3^ With a patient inclusion criterion of tumors with EGFRvIII expression as low as 6%, it is tempting to speculate that patients with low target expression would not have the same potential to benefit from treatment as those with a higher EGFRvIII percentage and target expression levels are of important consideration in future clinical trial design.

One of the most difficult challenges associated with CAR T cell therapy is the selection of an appropriate tumor antigen to target. Target antigens can be broadly classified as TSA, tumor associated (TAA) or Embryonic Cancer Germline Antigens.^35^ Therapeutic targeting of TSAs provides a greater level of safety as the specific expression of these antigens on malignant tissue reduces the likelihood of on-target/off-tumor targeting. An example of a TSA is EGFRvIII, a mutation in the Epidermal Growth Factor Receptor expressed in a subset of Glioblastoma tumors.^36^ Comparatively, TAAs are expressed at a low level on healthy tissues but have a higher level of expression on malignant tissues. Targeting these antigens can result in on-target/off-tumor toxicity.^37^ CAR T cell therapy has been greatly successful in the treatment of some types of hematological malignancies by targeting CD19,^38^ a TAA expressed on both healthy and malignant B cells. Germline antigens include targets such as IL-13Rα2^39^ and are frequently expressed on malignant cells but also demonstrate minimal expression on healthy adult tissue. Therapeutic targeting of an antigen which is also expressed on healthy tissue risks damage to other non-malignant cells in the body.

Published results of glioblastom-specific CAR T cell clinical trials have reported only transient antitumor responses in trials targeting several different antigens.^3–6^ Targeting of the high-affinity cytokine receptor IL-13 receptor α2 (IL-13Rα2), expressed on over 80% of glioblastoma tumors, caused reductions in IL-13Rα2-expressing tumor cells in two of three patients with few side effects.^40^ Similarly, CAR T cells targeting EGFRvIII in glioblastoma patients also resulted in reduced EGFRvIII-expressing tumor cells in five of seven patients. However, in both studies, an antigen-negative population remained, and this antigen escape allowed the majority of the tumor to persist, ultimately resulting in poor overall survival.^3 40^

While targeting TSAs in the context of solid tumors provides the greatest level of safety to avoid off tumor toxicity, the heterogeneous and potential low expression of TSAs has contributed to the lower success rate for CAR T cell therapy. However, using these antigens as induction signals of a logic gated system, rather than the target of therapy may provide greater anti-tumor function and more complete tumor cell elimination than what is induced by conventional CAR T treatment. Importantly, logic gating is still a form of targeted therapy and therefore, these systems are not immune from inducing target antigen downregulation or loss via antigen escape.

### Modulation of immune responses

The application of various logic gating systems has the potential to modulate immune responses. Depending on the type of system designed and the form of logic gating which is most suitable, immune responses such as CRS and off-tumor targeting of healthy tissue can be reduced, by the direct and local influence on tumor microenvironment.

CRS is another important consideration for CAR T cell therapy, particularly for hematological malignancies. The large quantity of proinflammatory cytokines such as IFN-γ systemically produced by highly activated CAR T cells activates bystander myeloid cells to release additional proinflammatory cytokines; including the potent IL-6.^41^ Therefore logic-gating the local secretion of anti-inflammatory mediators may assist in controlling systemic CRS.

Cases of CRS vary in severity, but the most severe cases can lead to multi-organ failure and death.^41^ The use of a monoclonal antibody (tocilizumab, α-IL-6) has shown efficacy in treating CRS without compromising CAR T cell efficacy.^41^ The availability of such anti-inflammatory medications is currently quite topical as tocilizumab is efficacious in treating patients infected with COVID-19.^42 43^ This has led to widespread redeployment of the medication for treating COVID-19 infections and consequently manufacturing delays and shortages.^44^ While this is an issue currently, and over time will resolve, the sudden reallocation of such medication further strengthens the case for approaches such as logic gating, whereby immune responses can be modulated without the external delivery of the drug itself, rather it can be produced locally by the infused logic gated T cell product.

Some degree of inflammatory cytokine release can enhance antitumor responses by activating bystander cells, however, a fine balance must be achieved to avoid excessive cytokine release and damage to tissues. Using logic gating systems, CRS could be controlled with the gated release of anti-inflammatory cytokines such as IL-10, monoclonal antibodies tocilizumab or mediators within the tumor microenvironment. This would mean that the tumor cells could still be eliminated, but the adverse patient responses of CRS may be avoided.

Logic gating approaches using inhibitory signaling pathways can further aid discrimination between healthy and malignant tissue. Genetic circuits built to only be induced in the absence of the ligation of a healthy antigen targeting CAR may prevent the off-tumor toxicity which can be observed when the primary target is minimally expressed on healthy cells. These types of circuits could prevent off-tumor toxicity before they occur. The application of inhibitory logic gating circuits may also work to increase the possible pool of target antigens by providing a checkpoint of ‘no healthy antigen’ on the cells before the destruction of the tumor cell, increasing safety.

Novel approaches are still needed to provide a greater level of regulation and control over engineered and infused T cells. Logic gating provides an additional level of control and safety and depending on the gating mechanism, even multi-antigen, or combination approaches. There are three main logic gating approaches described in this review and we will provide examples from the literature where they have been applied to engineered T cells and discuss some reflections on future approaches.

## Examples of biological logic gating

**Figure 2:**
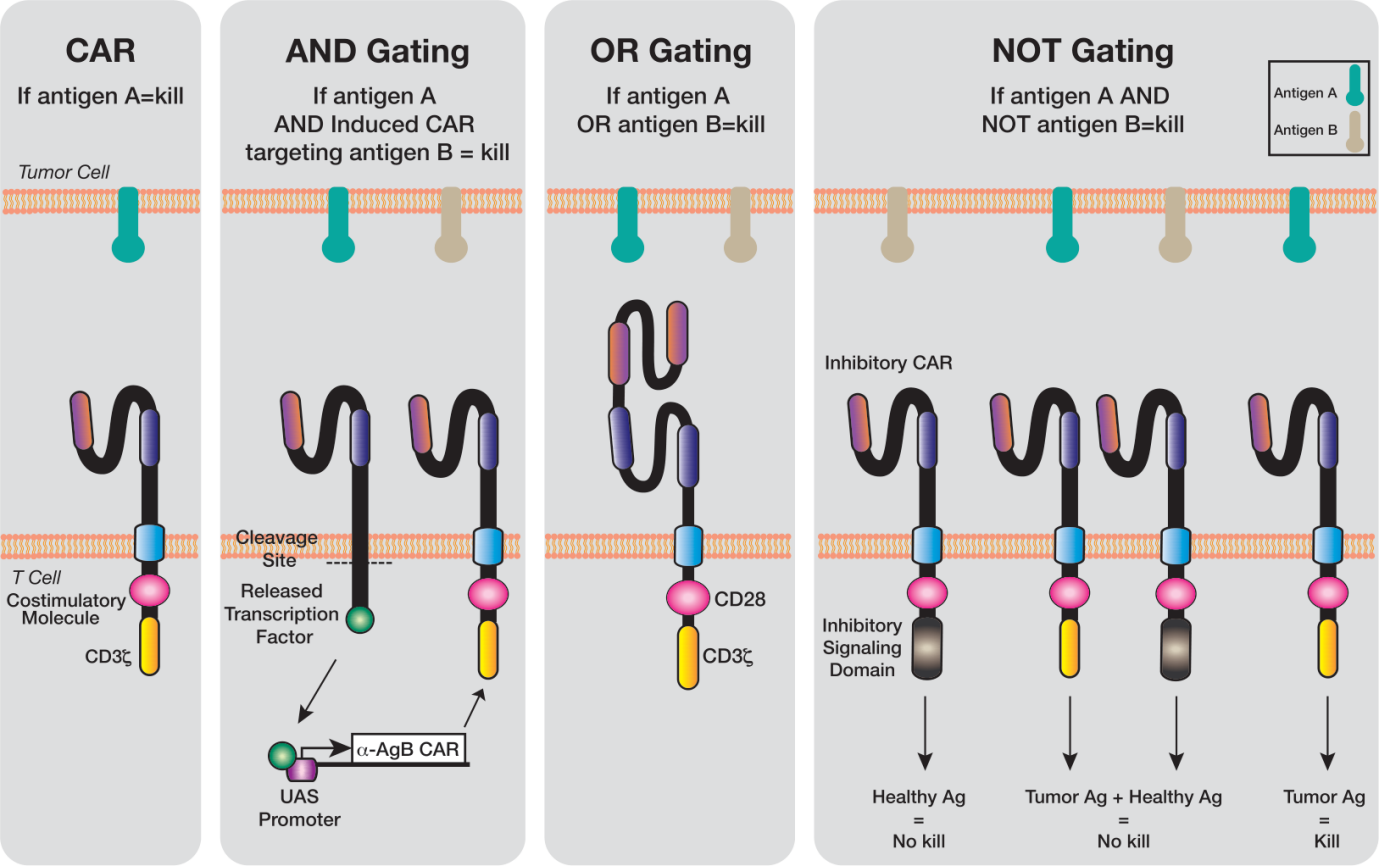
Logic gating technology has been applied to genetically engineered T cells. Chimeric Antigen Receptor (CAR) T cells bind to their target antigen and induce cell death. Using AND gating, genetic circuits are created where the ligation of the receptor induces cleavage of the transcription factor and induction of a second CAR targeting antigen B. CAR T cells created with an OR gate will kill the target cell in the presence of antigen A or B. Inhibitory cars use a NOT gating system and are designed with inhibitory signaling domains which if ligated, will not kill the target cell.

Logic gating techniques have begun to be incorporated into the design of engineered T cells. These systems allow either multi-antigen targeting, combinatorial treatment with biological agents (such as checkpoint inhibitors or cytokines) and potentially a level of safety that could not be achieved with the current generation of CAR T cells. There are multiple types of logic circuits which can be applied to genetically engineered T cells (figure 2). Biological AND-gating occurs when two input signals or events must occur before the outcome. In OR-gating, there may be multiple possible input signals and any of them can trigger the desired outcome. Finally, NOT- gating occurs when the signal input results in an inhibitory signal being generated, as opposed to the usual activation signal seen in each of the other logic gating circuits.

### AND-gating

One of the early forms of exerting control over engineered T cells was the development of ON switches. In this system, the administration of small molecules enables the completion of a circuit to allow T signalling.^45^ One such system was developed by Wu *et al*^46^ which they termed ON CARs.

In this system, the antigen-binding domain and the signaling domain (containing the CD3ζ domain and ITAMs) are two separate molecules that only associate in the presence of a small molecule. This small molecule is a modified version of rapamycin with reduced immunosuppressive activity,^47^ which the authors refer to as rapalog.^46^ In the presence of the target antigen and rapalog, the functional anti-CD19 CAR unit is formed; an example of AND-gating. An advantage of this system is that the level of functional CARs can be modulated by titration of rapalog, with an increased dose leading to a greater reduction in tumor burden in mouse models of leukemia treated with ON CAR T cells.^46^

One year after the publication of pharmacological control of ON switched CARs, the technology was enhanced by the ‘IF-THEN’ or AND logic gating system. The concept of AND-gating requires two distinct antigens to be detected to trigger the outcome or downstream event; one to induce the logic gated system, the other to kill the target cell. The benefit of genetic approaches such as AND-gating in biological systems results in the presence of potentially potent anti-tumor molecules being modulated within the tumor environment and thus limiting systemic expression which may be intolerable to patients.^24^

In 2016, the design of the ‘AND-gated’ Synthetic Notch receptor (SynNotch) was developed by Morsut *et al*.^14^ ^15^ In this system, the ligand binding domain is linked to the Notch core (a natural ligand in the body with a role in development^48^ within the transmembrane domain and a transcription factor. While there are multiple transcription factors which can be used in this system, the authors investigated the specific function of the Gal4-VP64 transcription factor in human cells. This system is an enhancement on the 2015 publication of ON switches by providing control over CAR expression, without the need for systemic delivery of recombinant antigen binding domains or small molecules.

On ligation of the scFv with its target antigen, the transcription factor Gal4-VP64 is cleaved from the SynNotch receptor by the natural Notch cleavage enzyme γ-secretase and the transcription factor is translocated into the nucleus of the cell where it becomes bound to the Upstream Activating Sequence. After binding, Gal4-VP64 drives the transcription of the desired cellular program, designed by the user (figure 2). In Roybal’s seminal 2016 publication, programs of cytokine production, monoclonal antibody production, transcription factors and even the expression of a second CAR were all shown to have the potential to be driven via SynNotch circuitry,^49^ providing evidence of a powerful circuit to regulate multiple anti-tumor mediators. Though one important characteristic of the system to consider is the inherent kinetic delay in the time from receptor ligation to transcription of the genetically coded signal (be that CAR expression, cytokine release, etc). Roybal *et al* noted an interval of approximately 6 hours from SynNotch activation to CAR expression (as determined by GFP expression of the tagged CAR).^15^ So while induction of the second protein may be slow, the extent of the kinetic delay may change with alternative induction antigens and future receptor designs.

The SynNotch system has been applied to multiple cancer models, including leukaemia,^49–51^ lung adenoarcinoma,^52^ mesothelioma,^51^ ovarian cancer^51^ and glioblastoma,^53^ in which tumor heterogeneity can be a contributing factor to unsuccessful targeted therapy. The glioblastoma tumor-specific protein EGFRvIII-SynNotch mediated induction of a Tandem CAR—a single CAR structure with dual targeting scFvs, specific for EphA2 and IL-13Rα2,^53^ was recently shown to induce more complete clearance of the glioblastoma orthotopic xenograft model GBM6 and result in longer survival compared with conventional single specific EGFRvIII, EphA2 or IL-13Rα2 CARs alone.^53^ Additionally, myelin oligodendrocyte glycoprotein was also used to mediate the induction of the EphA2 and IL-13Rα2 tandem CAR; thus providing evidence that normal antigens at the tumor site can also be used as induction signals in the SynNotch system.

The SynNotch system has also been applied to mesothelioma and ovarian cancer using the same ‘pan’ tumor antigen; ALPPL2, as the activation signal.^51^ The use of a more broadly applicable ‘pan’ cancer antigen was also investigated by Cho *et al*, who designed both a CAR and a SynNotch receptor specific for the receptor tyrosine kinase Axl^50^—expressed in breast, lung, colon and pancreatic cancers. The authors demonstrated the feasibility of this Axl system, achieving specific Axl-mediated CAR activation and induction of IL-10 on SynNotch activation.^50^ These studies have identified and characterized additional induction antigens which are amenable to the SynNotch system, both of which have the potential for a wide application to multiple cancer indications. The system can then be mixed and matched for the most efficacious genetic program to be induced (such as cytokines or additional CARs), dependent on the malignancy type and the tumor microenvironment to which it is applied.

Logic gating has also been shown to have a positive effect on T cell phenotype. There is evidence to suggest that the fine balance of signals, received by the T cell induces changes the differentiated state and indeed, tonic signaling induced by a CAR has been shown to influence T cell fate.^54 55^ Some early evidence has been published suggesting that logic-gating T cells can influence T cell fitness. In both the Hyrenius-Wittsten *et al*^51^ and Choe *et al*^53^ publications, the authors demonstrate logic gated T cells display a more ‘naïve’ like phenotype (CD62L^+^ CD45RA^+^) and reduced expression of checkpoint molecules. Hyrenius-Wittsten *et al*, propose that this less differentiated state is due to the removal of tonic signaling by changing the receptor design (compared with a conventional CAR).^51 53^ Hence, it is possible that logic gated technology may be a powerful tool to improve therapy efficacy by preserving the T cell population in a less differentiated state, while allowing modulation of the tumor microenvironment.

To date, Roybal *et al* have been the dominant investigators in the SynNotch research field, thus, the research field is small but growing. Other research groups have also applied SynNotch logic gating in their studies. Stanley Riddell’s group have developed an ‘AND’ gated ROR-1 specific CAR T cell which improved the safety of targeting this TAA by requiring the coexpression of another protein; EpCAM, or, for more tumor specificity B7-H3, preventing direction of cytotoxicity towards ROR-1 expressing stromal cells.^56^ Interestingly, protection of ROR-1 stromal cells was only found to occur when there was a spatial segregation between malignant and healthy cells. This paper nicely demonstrates a limitation of the SynNotch system, in that if healthy cells expressing a low level of the target antigen (TAA such as HER-2) are not spatially separated from the tumor cells, the healthy cells are likely to be destroyed by the T cell.^56^

The benefit of systems such as AND-gating broadens the applicability of many anti-tumor or immune activating molecules which may have been prevented from clinical use because of their systemic adverse effects. Stringent safety testing will be required to fully characterize the induction and half-life of the induced protein production. AND-gating is yet to be tested in clinical trials and so the ‘real world’ success is to be determined.

### OR-gating

The concept of OR-gating is an example of a multi-antigen approach requiring the recognition of either one or more of the targeted CAR T cell antigens (figure 2). This strategy expands the CAR T cell antigen repertoire and therefore increases the likelihood of tumor eradication for heterogeneous tumors. There are a variety of different approaches to the OR-gated CAR T cell concept, such as pooling a combination of single antigen targeting CAR T cells or engineering a single CAR receptor to target multiple antigens termed tan- or biCARs. CAR T cells can also be engineered to express CARs targeting two different antigens (dual CAR T), as well as three or more antigens such as triCARs or quad-CARs.

Multiantigen targeting CAR T cell capabilities have demonstrated enhanced anti-tumor elimination compared with single targeting strategies.^57^ The simplest of these strategies is the use of pooled CAR T cells, which involves combining two single-specific CAR T cell populations.^58^ This approach has been used in both hematological cancer models, by targeting CD19 and CD123 for the treatment of B-cell acute lymphoblastic leukemia (B-ALL)^58^ and in solid tumors such as glioblastoma by targeting both HER-2 and IL-13Rα2.^59^ In both studies, pooling two populations of single-specific CAR T cells significantly enhanced tumor elimination both in vitro and in in vivo mouse models, particularly when the cancer models heterogeneously expressed different antigens.

**Figure 3:**
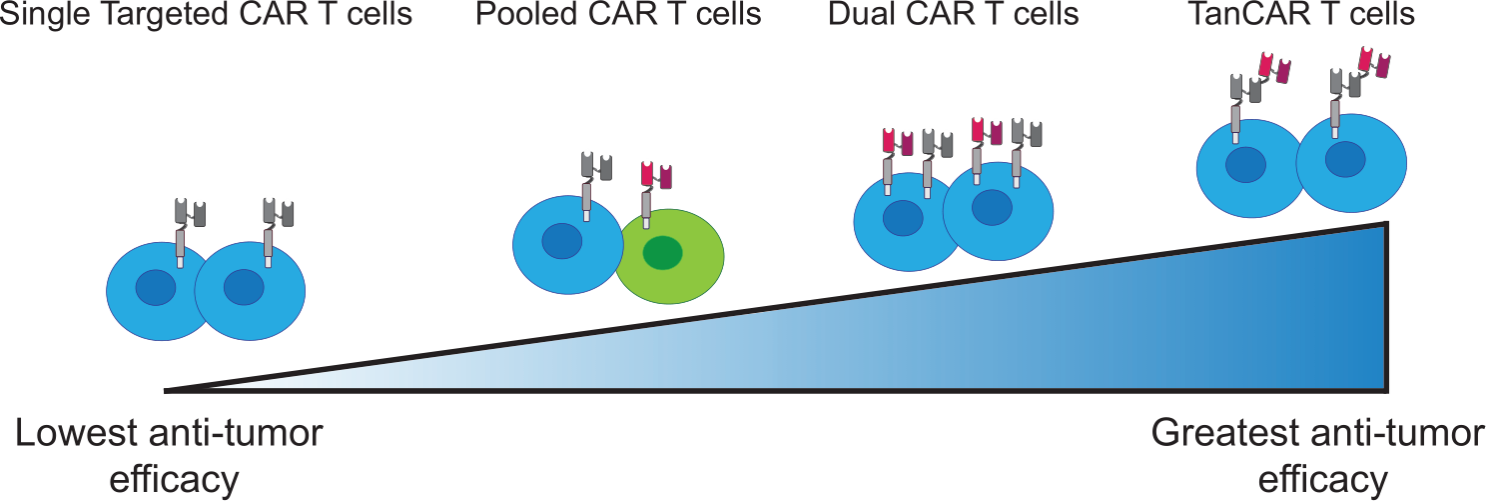
CAR modality effects anti-tumor function. Chimeric Antigen Receptor (CAR) T cells expressing a CAR specific for a single antigen demonstrate the lowest anti-tumor efficacy. Pooled CAR T cells are more efficacious than single specific CAR T cells and are generated by pooling two populations of single specific CAR T cells. Dual CAR T cells express multiple CARs on the one T cell. The most effective CAR modality is TanCAR T cells, with each T cell expressing one CAR engineered with multiple antigen recognition domains (scFvs). scFv, short-chain variable fragment.

Dual targeting antigens can be particularly advantageous to mitigate antigen escape or antigen loss. Clinically, CD19 antigen reduction or loss following treatment with CD19-targeted immunotherapies is often observed in relapsed and/or refractory B-ALL.^60^ This was shown to be mitigated by sequentially targeting a second antigen with maintained expression, using CD22 targeted CAR T cells, which resulted in 73% complete remission,^61^ though CD22-negative relapses were later observed.^62^ In addition, simultaneous antigen targeting by CD19/CD22 dual-specific CAR T cells, resulted in 88% complete remission.^62^ Several clinical trials using dual CAR T cells are currently ongoing.^61 63^ Furthermore, tandem CARs or tanCARs, in which a bispecific CAR receptor targets two different antigens (figure 3), has shown to further enhance tumor elimination,^64–66^ with a CD19/CD20 tanCAR clinical trial demonstrating promising results.^67^ In the treatment of solid tumors using a U373 glioblastoma model, Hegde *et al* demonstrated that tanCAR T cells controlled tumor growth significantly longer than pooled and dual CAR T cells.^59 64^

The benefit of the OR-gated designs of multiantigen targeted CAR T cells increases recognition capacity by CAR T cells and enables the genetic targeting of heterogeneous tumors, minimizing antigen escape. However, while these approaches offer hope to eliminate heterogeneous tumors, OR-gated designs have been associated with significant on-target/off-tumor adverse events. In studies of B-cell malignancies, the administration of dual CAR T cells resulted in significant CRS in multiple clinical trials, including CD19/CD22 dual CAR T cells,^68^ and CD19/CD20 tanCAR T cells.^67^ This demonstrates the importance of antigen target selection, and the use of additional safety measures, some of which have been engineered into the gated CAR T cell design, such as those using AND-gated or NOT-gated approaches.

### NOT-gating

Many proteins which are highly expressed on tumor cells, may also be expressed at low levels on healthy tissue. Whist the inclusion of suicide switches in genetically engineered cells provides a level of safety, this activation is irreversible and binary; either on or off. Once the suicide gene has been activated, the transduced cells are destroyed, no longer exerting anti-tumor functions, or providing benefit to the patient.

Unlike AND-circuits or OR-circuits which result in activation, the inhibitory NOT- logic gate prevents T cell activation on target recognition. NOT-gating can be applied to tumor antigens which may not be exquisitely tumor specific and display a low level of expression on healthy cells.

Inhibitory CARs (iCARs) are designed with inhibitory signaling domains such as those found in PD-1 and CTLA-4^69^ (well characterized T-cell inhibitory receptors). iCARs are used in combination with CARs specific for TAAs to reduce the likelihood of healthy tissue damage. Biological function (cytotoxic T cell responses) will only occur if the TAA-CAR AND-NOT- the healthy antigen CAR are ligated. In this way, the biological gating system is a combination of AND-NOT systems.

The function of iCARs has been evaluated in vitro and in vivo. Fedorov *et al* demonstrated in vitro that iCARs had greatly reduced cytokine production and cytotoxic capabilities when cocultured with induced pluripotent stem cell-derived differentiated into fibroblasts which displayed both healthy (PSMA) and tumor (CD19) antigens compared with levels measured with coculture with target antigen (CD19) expressing cells.^69^ To determine the functional capacity of the iCARs in vivo, the authors generated a NALM-6 model of leukemia, expressing both CD19 and the healthy antigen PSMA. Mice bearing tumors lacking PSMA expression showed a reduced tumor burden by bioluminescence imaging compared with mice bearing CD19+/PSMA +tumors when treated with the iCARs.^69^ Flow cytometric analysis of the bone marrow confirmed similar proportions of dual positive tumor cells remaining in iCAR treated mice compared with the non-T cell treated control. These results demonstrated the applicability of this system in vitro and in vivo. However, one critical consideration of this system is that both the iCAR and the target were required to be highly expressed for optimal suppression of CAR function,^69^ which is unlikely to be a possible combination for all tumor antigens.

Despite these promising findings, it is important to consider the potential implications of genetic circuits on T cell health and phenotype. The premise of the iCAR is that the inhibitory signal generated by ligation of a ‘healthy/normal protein’ by the iCAR will override any effector ‘kill’ signal induced by ligation of a ‘tumor targeting’ CAR. Therefore, the resulting T cell function is dependent on the balance of activation and inhibitory signals. Mackall *et al* have shown preliminary evidence to suggest that T cells expressing both the CD93 NOT-gated CAR T cells and CD19 iCAR T cells exhibited decreases in PD-1 and TIM-3 expression compared with mock transduced and CD93-28z CAR T cells at baseline, however, further phenotypic analysis on T cell fitness post iCAR activation was not investigated.^70^

Opposing most of the biological logic gating systems which promote T cell toxicity, there is another class of logic gating inhibiting T cell function. This family of receptors is referred to as OFF-switch. OFF-switches are generally pharmacologically mediated.^45 71 72^ One example of the OFF-switch system is the Chemically Regulated-SH2-delivered Inhibitory Tail (CRASH-IT). These small molecule mediated switches can be applied both to engineered CAR T cells but also within the T cell receptor (TCR) complex. The switch was composed of an SH-2 binding domain fused to the intracellular signaling domain of PD-1 to inhibit signaling. Degradation of the inhibitory complex was achieved via the creation of a fusion protein containing a protease and a degron (a protein component targeting it for degradation by the proteosome). The benefit of the CRASH-IT switch is that it can be repeatably activated without resulting in engineered cell destruction.^73^

The authors also showed titratable function of the switch using a small molecule; asunaprevir (an anti-viral drug approved for use in combination with Daclatasvir in the treatment of Hepatitis C.^74^ In this study, in the absence of asunaprevir, the link between the degron and the protein of interest was degraded. However, the presence of the small molecule held the entire complex together, targeting it for degradation. This provides the same benefit of a suicide switch in preventing activation of the system, but in a non-binary manner and allowing reactivation of the system. While an excellent proof of principle, whether or not this finding can be recapitulated in vivo is yet to be reported.

Inhibitory circuits, or NOT-gates, provide an opportunity for the field to begin to investigate the anti-tumor function of therapies targeting highly expressed tumor antigens, which may have some level of expression on healthy tissue. The inbuilt safety step of either triggering an inhibitory signaling event or an inhibitory signaling event and degradation of the CAR could provide some level of reassurance that any targeting of healthy tissue can be halted before tissue destruction occurs.

## Conclusions

Genetically engineered T cells have revolutionized the treatment for many types of hematological malignancies, improving both progression-free and overall survival. However, the same success is yet to be translated to solid tumors.^3 4^ The treatment of solid tumors with precision medicines can often result in antigen escape due to numerous factors including tumor heterogeneity (both interpersonal and intratumoral) and level of antigen expression required for effective function of infused T cells. The field of oncology is moving towards combination treatments, but progress in the field of genetic engineering may mean that we can deliver the benefits of multiantigen targeting in a single, targeted treatment.

Approaches such as biological logic gating enable a greater level of safety to be achieved with engineered T cells. The anti-tumor payload in the form of cytokines or additional CARs can be delivered directly to the tumor site and further finetuning of molecules such as with OFF switches, pharmacological modification or NOT-gating may reduce the likelihood of leakiness out of the tumor microenvironment. Restricting expression of these molecules locally within the tumor microenvironment broadens the applicability of many anti-tumor therapeutics.

There may be limitless possibilities when it comes to designing a logic gating system. Wendell Lim’s group has published on multiple effective logic gates; AND, NOT, Multiantigen AND gates, OR and even OFF-Notch; logic gating used to induce the expression of apoptotic factors within the cell.^75^ These logic gated systems could be directed to recognize both extracellular antigens and peptide:MHC complexes. Furthermore, the authors built into the systems the requirement for two or three antigens to induce the logic gated response, in an OR-AND circuit.^75^ Jan, Maus and colleagues describe a drug dependent ON/OFF switch system using degron tags which was combined with a split CAR.^45^ These publications demonstrate the flexibility of using logic gating and the multitude of possible applications including multiple types of gating within the one system.

However, despite these advantages, logic gated systems have not yet made a clinical appearance. Publications by field leaders Roybal and Lim have demonstrated efficacy both in vitro and in vivo for numerous logic gated systems but currently, no clinical trials have been published. Depending on the nature of the induced molecule, an in-depth understanding of the expression and degradation kinetics will be required before progression to in-person trials.

CAR T cells and T cell-based therapeutics form a critical component of immunotherapy approaches. Enhanced engineering of these systems is likely to enhance safety and potential efficacy against solid tumors by providing a multi-targeted approach directly at the tumor site. Early mechanisms of control overengineered cells include suicide switches which result in the irreversible destruction of the engineered cell. Advances in genetic engineering have resulted in the development of a range of logic gated systems. These approaches provide multiple benefits to engineered cells, including enhanced control and safety, with potential protective mechanisms against on-target/off-tumor toxicity and CRS. While a greater level of understanding of induction and degradation kinetics is still required and in patient trials, this technology has the potential to enhance patient responses to CAR T cell therapy in treating solid tumors.

## References

[1] Zhao Z, Condomines M, van der Stegen SJC, et al. Structural design of engineered costimulation determines tumor rejection kinetics and persistence of CAR T cells. Cancer Cell 2015;28:415–28. 10.1016/j.ccell.2015.09.004 26461090 PMC5003056

[2] Maude SL, Frey N, Shaw PA, et al. Chimeric antigen receptor T cells for sustained remissions in leukemia. New England Journal of Medicine 2014;371:1507–17. 10.1056/NEJMoa1407222 PMC4267531 25317870

[3] O’Rourke DM, Nasrallah MP, Desai A, et al. A single dose of peripherally infused EGFRvIII-directed CAR T cells mediates antigen loss and induces adaptive resistance in patients with recurrent glioblastoma. Sci Transl Med 2017;9. 10.1126/scitranslmed.aaa0984 PMC5762203 28724573

[4] Goff SL, Morgan RA, Yang JC, et al. Pilot trial of adoptive transfer of chimeric antigen Receptor–transduced T cells targeting EGFRvIII in patients with glioblastoma. J Immunother 2019;42:126–35. 10.1097/CJI.0000000000000260 30882547 PMC6691897

[5] Brown CE, Alizadeh D, Starr R, et al. Regression of glioblastoma after chimeric antigen receptor T-cell therapy. N Engl J Med 2016;375:2561–9. 10.1056/NEJMoa1610497 28029927 PMC5390684

[6] Ahmed N, Brawley V, Hegde M, et al. Her2-Specific chimeric antigen receptor-modified virus-specific T cells for progressive glioblastoma: a phase 1 dose-escalation trial. JAMA Oncol 2017;3:1094–101. 10.1001/jamaoncol.2017.0184 28426845 PMC5747970

[7] Joyce JA, Fearon DT, Joyce Johanna A. T cell exclusion, immune privilege, and the tumor microenvironment. Science 2015;348:74–80. 10.1126/science.aaa6204 25838376

[8] Newick K, O'Brien S, Moon E, et al. Car T cell therapy for solid tumors. Annu Rev Med 2017;68:139–52. 10.1146/annurev-med-062315-120245 27860544

[9] Hughes-Parry HE, Cross RS, Jenkins MR. The evolving protein engineering in the design of chimeric antigen receptor T cells. Int J Mol Sci 2019;21:204. 10.3390/ijms21010204 PMC6981602 31892219

[10] Marin V, Cribioli E, Philip B, et al. Comparison of different suicide-gene strategies for the safety improvement of genetically manipulated T cells. Hum Gene Ther Methods 2012;23:376–86. 10.1089/hgtb.2012.050 23186165 PMC4015080

[11] Di Stasi A, Tey S-K, Dotti G, et al. Inducible apoptosis as a safety switch for adoptive cell therapy. N Engl J Med Overseas Ed 2011;365:1673–83. 10.1056/NEJMoa1106152 PMC3236370 22047558

[12] Lee SM, Kang CH, Choi SU, et al. A chemical switch system to modulate chimeric antigen receptor T cell activity through proteolysis-targeting chimaera technology. ACS Synth Biol 2020;9:987–92. 10.1021/acssynbio.9b00476 32352759

[13] Tiberghien P et al. Administration of herpes simplex-thymidine kinase-expressing donor T cells with a T-cell-depleted allogeneic marrow graft. Blood 2001;97:63–72. 10.1182/blood.V97.1.63 11133743

[14] Morsut L, Roybal KT, Xiong X, et al. Engineering customized cell sensing and response behaviors using synthetic Notch receptors. Cell 2016;164:780–91. 10.1016/j.cell.2016.01.012 26830878 PMC4752866

[15] Roybal KT, Rupp LJ, Morsut L, et al. Precision tumor recognition by T cells with combinatorial Antigen-Sensing circuits. Cell 2016;164:770–9. 10.1016/j.cell.2016.01.011 26830879 PMC4752902

[16] Lajoie MJ, Boyken SE, Salter AI, et al. Designed protein logic to target cells with precise combinations of surface antigens. Science 2020;369:1637–43. 10.1126/science.aba6527 32820060 PMC8085813

[17] Chmielewski M, Kopecky C, Hombach AA, et al. Il-12 release by engineered T cells expressing chimeric antigen receptors can effectively Muster an antigen-independent macrophage response on tumor cells that have shut down tumor antigen expression. Cancer Res 2011;71:5697–706. 10.1158/0008-5472.CAN-11-0103 21742772

[18] Chmielewski M, Abken H. Trucks: the fourth generation of cars. Expert Opin Biol Ther 2015;15:1145–54. 10.1517/14712598.2015.1046430 25985798

[19] Chmielewski M, Abken H. Trucks, the fourth‐generation CAR T cells: current developments and clinical translation. Advances In Cell And Gene Therapy 2020;3. 10.1002/acg2.84

[20] Doi K, Imai T, Kressler C, et al. Crucial role of the Rap G protein signal in Notch activation and leukemogenicity of T-cell acute lymphoblastic leukemia. Sci Rep 2015;5:7978 10.1038/srep07978 25613394 PMC4303867

[21] Barrett JA, Cai H, Miao J, et al. Regulated intratumoral expression of IL-12 using a RheoSwitch therapeutic System® (RTS®) gene switch as gene therapy for the treatment of glioma. Cancer Gene Ther 2018;25:106–16. 10.1038/s41417-018-0019-0 29755109 PMC6021367

[22] Caruso HG, Heimberger AB, Cooper LJN. Steering CAR T cells to distinguish Friend from foe. Oncoimmunology 2019;8:e1271857. 10.1080/2162402X.2016.1271857 31646067 PMC6791456

[23] Agliardi G, Liuzzi AR, Hotblack A, et al. Intratumoral IL-12 delivery empowers CAR-T cell immunotherapy in a pre-clinical model of glioblastoma. Nat Commun 2021;12:444. 10.1038/s41467-020-20599-x 33469002 PMC7815781

[24] Leonard JP, Sherman ML, Fisher GL, et al. Effects of single-dose interleukin-12 exposure on interleukin-12-associated toxicity and interferon-gamma production. Blood 1997;90:2541–8. 9326219

[25] Lai J, Mardiana S, House IG, et al. Adoptive cellular therapy with T cells expressing the dendritic cell growth factor Flt3L drives epitope spreading and antitumor immunity. Nat Immunol 2020;21:914–26. 10.1038/s41590-020-0676-7 32424363

[26] Kuhn NF, Purdon TJ, van Leeuwen DG, et al. Cd40 Ligand-Modified chimeric antigen receptor T cells enhance antitumor function by eliciting an endogenous antitumor response. Cancer Cell 2019;35:e476:473–88. 10.1016/j.ccell.2019.02.006 PMC6428219 30889381

[27] Grupp SA, Maude SL, Shaw PA, et al. Durable remissions in children with relapsed/refractory all treated with T cells engineered with a CD19-Targeted chimeric antigen receptor (CTL019). Blood 2015;126:681. 10.1182/blood.V126.23.681.681

[28] Ruella M, Maus MV. Catch me if you can: leukemia escape after CD19-Directed T cell immunotherapies. Comput Struct Biotechnol J 2016;14:357–62. 10.1016/j.csbj.2016.09.003 27761200 PMC5061074

[29] Bedard PL, Hansen AR, Ratain MJ, et al. Tumour heterogeneity in the clinic. Nature 2013;501:355–64. 10.1038/nature12627 24048068 PMC5224525

[30] Perez K, Walsh R, Brilliant K, et al. Heterogeneity of colorectal cancer (CRC) in reference to KRAS proto-oncogene utilizing wave technology. Exp Mol Pathol 2013;95:74–82. 10.1016/j.yexmp.2013.01.004 23528430 PMC4015467

[31] Yancovitz M, Litterman A, Yoon J, et al. Intra- and inter-tumor heterogeneity of BRAF(V600E))mutations in primary and metastatic melanoma. PLoS One 2012;7:e29336. 10.1371/journal.pone.0029336 22235286 PMC3250426

[32] Sakurada A, Lara-Guerra H, Liu N, et al. Tissue heterogeneity of EGFR mutation in lung adenocarcinoma. Journal of Thoracic Oncology 2008;3:527–9. 10.1097/JTO.0b013e318168be93 18449007

[33] Tougeron D, Lecomte T, Pagès JC, et al. Effect of low-frequency KRAS mutations on the response to anti-EGFR therapy in metastatic colorectal cancer. Annals of Oncology 2013;24:1267–73. 10.1093/annonc/mds620 23293113

[34] He D, Wang D, Lu P, et al. Single-Cell RNA sequencing reveals heterogeneous tumor and immune cell populations in early-stage lung adenocarcinomas harboring EGFR mutations. Oncogene 2021;40:355–68. 10.1038/s41388-020-01528-0 33144684 PMC7808940

[35] Abbott RC, Cross RS, Jenkins MR. Finding the keys to the CAR: identifying novel target antigens for T cell redirection immunotherapies. Int J Mol Sci 2020;21:515. 10.3390/ijms21020515 PMC7014258 31947597

[36] Schwechheimer K, Huang S, Cavenee WK. EGFR gene amplification--rearrangement in human glioblastomas. Int J Cancer 1995;62:145–8. 10.1002/ijc.2910620206 7622287

[37] Morgan RA, Yang JC, Kitano M, et al. Case report of a serious adverse event following the administration of T cells transduced with a chimeric antigen receptor recognizing ErbB2. Mol Ther 2010;18:843–51. 10.1038/mt.2010.24 20179677 PMC2862534

[38] Porter DL, Levine BL, Kalos M, et al. Chimeric antigen receptor-modified T cells in chronic lymphoid leukemia. N Engl J Med 2011;365:725–33. 10.1056/NEJMoa1103849 21830940 PMC3387277

[39] Debinski W, Gibo DM, Hulet SW, et al. Receptor for interleukin 13 is a marker and therapeutic target for human high-grade gliomas. Clin Cancer Res 1999;5:985–90. 10353730

[40] Brown CE, Badie B, Barish ME, et al. Bioactivity and safety of IL13Rα2-Redirected chimeric antigen receptor CD8+ T cells in patients with recurrent glioblastoma. Clin Cancer Res 2015;21:4062. 10.1158/1078-0432.CCR-15-0428 26059190 PMC4632968

[41] Maude SL, Barrett D, Teachey DT, et al. Managing cytokine release syndrome associated with novel T cell-engaging therapies. Cancer J 2014;20:119–22. 10.1097/PPO.0000000000000035 24667956 PMC4119809

[42] Toniati P, Piva S, Cattalini M, et al. Tocilizumab for the treatment of severe COVID-19 pneumonia with hyperinflammatory syndrome and acute respiratory failure: a single center study of 100 patients in Brescia, Italy. Autoimmun Rev 2020;19:102568. 10.1016/j.autrev.2020.102568 32376398 PMC7252115

[43] de Cáceres C, Martínez R, Bachiller P, et al. The effect of tocilizumab on cytokine release syndrome in COVID-19 patients. Pharmacol Rep 2020;72:1529–37. 10.1007/s43440-020-00186-z 33165762 PMC7650573

[44] Verma AA, Pai M, Saha S, et al. Managing drug shortages during a pandemic: tocilizumab and COVID-19. CMAJ 2021;193:E771–6. 10.1503/cmaj.210531 33952621 PMC8177913

[45] Jan M, Scarfò I, Larson RC, et al. Reversible on- and OFF-switch chimeric antigen receptors controlled by lenalidomide. Sci Transl Med 2021;13:eabb6295. 10.1126/scitranslmed.abb6295 33408186 PMC8045771

[46] Wu C-Y, Roybal KT, Puchner EM, et al. Remote control of therapeutic T cells through a small molecule-gated chimeric receptor. Science 2015;350:aab4077 10.1126/science.aab4077 26405231 PMC4721629

[47] Liberles SD, Diver ST, Austin DJ, et al. Inducible gene expression and protein translocation using nontoxic ligands identified by a mammalian three-hybrid screen. Proc Natl Acad Sci U S A 1997;94:7825–30. 10.1073/pnas.94.15.7825 9223271 PMC21513

[48] Lowell S, Benchoua A, Heavey B, et al. Notch promotes neural lineage entry by pluripotent embryonic stem cells. PLoS Biol 2006;4:e121. 10.1371/journal.pbio.0040121 16594731 PMC1431581

[49] Roybal KT, Williams JZ, Morsut L, et al. Engineering T cells with customized therapeutic response programs using synthetic Notch receptors. Cell 2016;167:e416:419–32. 10.1016/j.cell.2016.09.011 PMC5072533 27693353

[50] Cho JH, Okuma A, Al-Rubaye D, et al. Engineering Axl specific CAR and SynNotch receptor for cancer therapy. Sci Rep 2018;8:3846. 10.1038/s41598-018-22252-6 29497107 PMC5832765

[51] Hyrenius-Wittsten A, Su Y, Park M, et al. SynNotch CAR circuits enhance solid tumor recognition and promote persistent antitumor activity in mouse models. Sci Transl Med 2021;13:eabd8836. 10.1126/scitranslmed.abd8836 33910981 PMC8594452

[52] Xia M, Chen J, Meng G, et al. Cxcl10 encoding synNotch T cells enhance anti-tumor immune responses without systemic side effect. Biochem Biophys Res Commun 2021;534:765–72. 10.1016/j.bbrc.2020.11.002 33213838

[53] Choe JH, Watchmaker PB, Simic MS, et al. SynNotch-CAR T cells overcome challenges of specificity, heterogeneity, and persistence in treating glioblastoma. Sci Transl Med 2021;13:eabe7378. 10.1126/scitranslmed.abe7378 33910979 PMC8362330

[54] Long AH, Haso WM, Shern JF, et al. 4-1Bb costimulation ameliorates T cell exhaustion induced by tonic signaling of chimeric antigen receptors. Nat Med 2015;21:581–90. 10.1038/nm.3838 25939063 PMC4458184

[55] Lynn RC, Weber EW, Sotillo E, et al. C-Jun overexpression in car T cells induces exhaustion resistance. Nature 2019;576:293–300. 10.1038/s41586-019-1805-z 31802004 PMC6944329

[56] Srivastava S, Salter AI, Liggitt D, et al. Logic-Gated Ror1 chimeric antigen receptor expression rescues T cell-mediated toxicity to normal tissues and enables selective tumor targeting. Cancer Cell 2019;35:e488:489–503. 10.1016/j.ccell.2019.02.003 PMC6450658 30889382

[57] Cui W, Zhang X, Dai H, et al. Tandem CD19/CD22 dual targets CAR-T cells therapy acquires superior Cr rate than CD19 CAR-T cells: a case controlled study. Blood 2020;136:44. 10.1182/blood-2020-143474

[58] Ruella M, Barrett DM, Kenderian SS, et al. Dual CD19 and CD123 targeting prevents antigen-loss relapses after CD19-directed immunotherapies. J Clin Invest 2016;126:3814–26. 10.1172/JCI87366 27571406 PMC5096828

[59] Hegde M, Corder A, Chow KKH, et al. Combinational targeting offsets antigen escape and enhances effector functions of adoptively transferred T cells in glioblastoma. Mol Ther 2013;21:2087–101. 10.1038/mt.2013.185 23939024 PMC3831041

[60] Maude SL, Teachey DT, Rheingold SR, et al. Sustained remissions with CD19-specific chimeric antigen receptor (CAR)-modified T cells in children with relapsed/refractory ALL. Journal of Clinical Oncology 2016;34:3011 10.1200/JCO.2016.34.15_suppl.3011

[61] Spiegel JY, Patel S, Muffly L, et al. Car T cells with dual targeting of CD19 and CD22 in adult patients with recurrent or refractory B cell malignancies: a phase 1 trial. Nat Med 2021;27:1419–31. 10.1038/s41591-021-01436-0 34312556 PMC8363505

[62] Fry TJ, Shah NN, Orentas RJ, et al. CD22-targeted CAR T cells induce remission in B-ALL that is naive or resistant to CD19-targeted CAR immunotherapy. Nat Med 2018;24:20–8. 10.1038/nm.4441 29155426 PMC5774642

[63] Shah NN, Johnson BD, Schneider D, et al. Bispecific anti-CD20, anti-CD19 CAR T cells for relapsed B cell malignancies: a phase 1 dose escalation and expansion trial. Nat Med 2020;26:1569–75. 10.1038/s41591-020-1081-3 33020647

[64] Hegde M, Mukherjee M, Grada Z, et al. Tandem CAR T cells targeting HER2 and IL13Rα2 mitigate tumor antigen escape. J Clin Invest 2016;126:3036–52. 10.1172/JCI83416 27427982 PMC4966331

[65] Grada Z, Hegde M, Byrd T, et al. TanCAR: a novel bispecific chimeric antigen receptor for cancer immunotherapy. Mol Ther Nucleic Acids 2013;2:e105. 10.1038/mtna.2013.32 23839099 PMC3731887

[66] Zah E, Lin M-Y, Silva-Benedict A, et al. T cells expressing CD19/CD20 bispecific chimeric antigen receptors prevent antigen escape by malignant B cells. Cancer Immunol Res 2016;4:498–508. 10.1158/2326-6066.CIR-15-0231 27059623 PMC4933590

[67] Tong C, Zhang Y, Liu Y, et al. Optimized tandem CD19/CD20 CAR-engineered T cells in refractory/relapsed B-cell lymphoma. Blood 2020;136:1632–44. 10.1182/blood.2020005278 32556247 PMC7596761

[68] Hossain N, Sahaf B, Abramian M, et al. Phase I experience with a bi-specific CAR targeting CD19 and CD22 in adults with B-cell malignancies. Blood 2018;132:490. 10.1182/blood-2018-99-110142

[69] Fedorov VD, Themeli M, Sadelain M. PD-1- and CTLA-4-based inhibitory chimeric antigen receptors (iCARs) divert off-target immunotherapy responses. Sci Transl Med 2013;5:ra172ra172. 10.1126/scitranslmed.3006597 PMC4238416 24337479

[70] Richards RM, Zhao F, Freitas KA, et al. NOT-Gated CD93 CAR T cells effectively target AML with minimized endothelial cross-reactivity. Blood Cancer Discov 2021;2:648–65. 10.1158/2643-3230.BCD-20-0208 34778803 PMC8580619

[71] Mestermann K, Giavridis T, Weber J, et al. The tyrosine kinase inhibitor dasatinib acts as a pharmacologic on/off switch for CAR T cells. Sci Transl Med 2019;11:eaau5907. 10.1126/scitranslmed.aau5907 31270272 PMC7523030

[72] Sommer C, Cheng H-Y, Nguyen D, et al. Allogeneic FLT3 CAR T cells with an Off-Switch exhibit potent activity against AML and can be depleted to expedite bone marrow recovery. Mol Ther 2020;28:2237–51. 10.1016/j.ymthe.2020.06.022 32592688 PMC7544976

[73] Sahillioglu AC, Toebes M, Apriamashvili G, et al. CRASH-IT switch enables reversible and dose-dependent control of TCR and CAR T-cell function. Cancer Immunol Res 2021;9:999–1007. 10.1158/2326-6066.CIR-21-0095 34193461 PMC8974419

[74] Manns M, Pol S, Jacobson IM, et al. All-oral daclatasvir plus asunaprevir for hepatitis C virus genotype 1B: a multinational, phase 3, multicohort study. The Lancet 2014;384:1597–605. 10.1016/S0140-6736(14)61059-X 25078304

[75] Williams JZ, Allen GM, Shah D, et al. Precise T cell recognition programs designed by transcriptionally linking multiple receptors. Science 2020;370:1099–104. 10.1126/science.abc6270 33243890 PMC8054651

[76] Cho JH, Collins JJ, Wong WW. Universal chimeric antigen receptors for multiplexed and logical control of T cell responses. Cell 2018;173:e1411:1426–38. 10.1016/j.cell.2018.03.038 PMC5984158 29706540

[77] Cartellieri M, Feldmann A, Koristka S, et al. Switching CAR T cells on and off: a novel modular platform for retargeting of T cells to AML blasts. Blood Cancer J 2016;6:e458 10.1038/bcj.2016.61 27518241 PMC5022178

[78] Ma JSY KJY, Kazane SA, S-h C. Versatile strategy for controlling the specificity and activity of engineered T cells. Proceedings of the National Academy of Sciences 2016;113:E450. 10.1073/pnas.1524193113 PMC4743826 26759368

[79] Tamada K, Geng D, Sakoda Y, et al. Redirecting gene-modified T cells toward various cancer types using tagged antibodies. Clinical Cancer Research 2012;18:6436–45. 10.1158/1078-0432.CCR-12-1449 23032741

[80] Urbanska K, Lanitis E, Poussin M, et al. A universal strategy for adoptive immunotherapy of cancer through use of a novel T-cell antigen receptor. Cancer Res 2012;72:1844–52. 10.1158/0008-5472.CAN-11-3890 22315351 PMC3319867

[81] Han X, Bryson PD, Zhao Y, et al. Masked chimeric antigen receptor for tumor-specific activation. Molecular Therapy 2017;25:274–84. 10.1016/j.ymthe.2016.10.011 28129121 PMC5363190

[82] Rodgers DT, Mazagova M, Hampton EN, et al. Switch-mediated activation and retargeting of CAR-T cells for B-cell malignancies. Proc Natl Acad Sci U S A 2016;113:E459–68. 10.1073/pnas.1524155113 26759369 PMC4743815

[83] Kudo K, Imai C, Lorenzini P, et al. T lymphocytes expressing a CD16 signaling receptor exert antibody-dependent cancer cell killing. Cancer Res 2014;74:93–103. 10.1158/0008-5472.CAN-13-1365 24197131

[84] Lanitis E, Poussin M, Klattenhoff AW, et al. Chimeric antigen receptor T cells with dissociated signaling domains exhibit focused antitumor activity with reduced potential for toxicity in vivo. Cancer Immunol Res 2013;1:43–53. 10.1158/2326-6066.CIR-13-0008 24409448 PMC3881605

[85] Kloss CC, Condomines M, Cartellieri M, et al. Combinatorial antigen recognition with balanced signaling promotes selective tumor eradication by engineered T cells. Nat Biotechnol 2013;31:71–5. 10.1038/nbt.2459 23242161 PMC5505184

